# The effects of Mediterranean diet on cardiovascular risk factors, glycemic control and weight loss in patients with type 2 diabetes: a meta-analysis

**DOI:** 10.1186/s40795-024-00836-y

**Published:** 2024-04-19

**Authors:** Xing Zheng, Wenwen Zhang, Xiaojuan Wan, Xiaoyan Lv, Peng Lin, Shucheng Si, Fuzhong Xue, Aijun Wang, Yingjuan Cao

**Affiliations:** 1 Department of Medical Oncology, Qilu Hospital, Cheeloo College of Medicine, Shandong University, Jinan, Shandong 250012 P.R. China; 2 Department of Thoracic Surgery, Qilu Hospital, Cheeloo College of Medicine, Shandong University, Jinan, Shandong 250012 P.R. China; 3 School of Nursing, Yangzhou University, Yangzhou, Jiangsu 225000 P. R. China; 4 Department of Nursing, Qilu Hospital, Cheeloo College of Medicine, Shandong University, Jinan, Shandong 250012 P. R. China; 5 Department of Endocrinology, Qilu Hospital, Cheeloo College of Medicine, Shandong University, Jinan, Shandong 250012 P. R. China; 6 Institute for Medical Dataology, School of Public Health, Cheeloo College of Medicine, Shandong University, Jinan, Shandong 250012 P. R. China; 7 Theory & Practice Innovation Research Center, Qilu Hospital, Cheeloo College of Medicine, Shandong University, Jinan, Shandong 250012 P. R. China

**Keywords:** Mediterranean diet, Type 2 diabetes, Ardiovascular risk factors, Glycemic control, Weight loss, Meta-analysis

## Abstract

To explore the impact of the Mediterranean diet on cardiovascular risk factors, glycemic control and weight loss in patients with type 2 diabetes(T2D) by a meta-analysis of randomized controlled trials (RCTs). We systematically searched PubMed, Cochrance Library, EMBASE and four Chinese databases to identify RCTs that compared the Mediterranean diet with control diets in patients with T2D up to December 2021. The Risk of Bias of the included studies was assessed using the version 2 of the Cochrane risk-of-bias tools for randomized trials (ROB 2). Seven RCTs with 1371 patients met the eligibility criteria and entered into the meta-analysis. Compared to control diets, the beneficial effects of Mediterranean diet were not statistically significant in high-density lipoprotein (MD = 2.33; 95% *CI*: -0.27 to 4.92), low-density lipoprotein (MD = -2.34; 95% CI -5.67 to 0.99) and total cholesterol (MD = 2.60; 95% CI: -0.95 to 6.15). But Mediterranean diet led to reduce the level of diastolic blood pressure (MD = -1.20; 95% CI: -2.21 to -0.19) and systolic blood pressure (MD = -4.17; 95% CI: -7.12 to -1.22). Meanwhile, Mediterranean diet showed beneficial effects in glycemic control (HbA1[%]: MD = -0.39, 95% CI: -0.58 to -0.20; fasting plasma glucose: MD = -15.12, 95% CI: -24.69 to -5.55) and weight loss (BMI: MD = -0.71, 95% CI: -1.30 to -0.78; WC: MD = -1.69; 95% CI: -3.35 to -0.02) compared to the control diets. The meta-analysis presented evidence supporting the beneficial effects of the Mediterranean diet on blood pressure, glycemic control, and weight loss. However, the impact of the Mediterranean diet on the lipid profile was not found to be significant, warranting further verification. This Meta-analysis was registered on the INPLASY website (Registration number: INPLASY 202160096).

**Supplementary Information:**

The online version contains supplementary material available at 10.1186/s40795-024-00836-y.

## Background

Type 2 diabetes ( T2D ) is a global disease. According to the survey, as of 2017, the number of patients with T2D in the world was as high as 425 million, and the number of people who die of diabetes each year was as high as 4 million [1]. In addition, the incidence rate is gradually increasing. Experts predicted that by 2035, the number of people with diabetes will reach 592 million [2], which will pose great threat to human health and cause huge medical economic costs. T2D is mainly characterized by elevated blood glucose, insulin resistance and decreased insulin sensitivity [3]. Investigation shows that the proportion of overweight or obese people in type 2 diabetic population is as high as 90% [4]. Most diabetic patients are accompanied by metabolic syndrome such as elevated blood lipid and blood pressure, which will increase the risk of various heart diseases [5]. Therefore, it is necessary to take effective measures for comprehensive management of T2D, and it is particularly important to control blood glucose and body weight to reduce the risk of heart disease. Lifestyle interventions have been recognized by experts and scholars as an effective means for the treatment of T2D and dietary changes can effectively improve insulin sensitivity, prevent the progression of the disease and delay the occurrence of complications [6, 7]. Current studies have shown that dietary patterns such as low fat [8], low carbohydrate [9], and high monounsaturated fatty acids [10] have certain effects on blood glucose control and weight of diabetic patients, but the results are not consistent. And the American Diabetes Association stated that there was not enough evidence to recommend the correct proportion of fat, protein and carbohydrate in the diet for patients with T2D [11].

The idea that Mediterranean diet is beneficial to human health has gradually been accepted by scholars around the world. Mediterranean diet, first proposed by Ancel Key in 1960 [12], is a unique diet of people living in the Mediterranean region. The Mediterranean diet is characterized by a high proportion of vegetables, fruits, beans, seafood and nuts. Vegetable oil (mainly olive oil, rich in unsaturated fatty acids) should be used for cooking, and a low proportion of red meat and processed foods should be used, as well as red wine with each meal [13–15]. A meta-analysis in 2015 demonstrated that, in comparison with other diets, the Mediterranean diet is more effective in controlling blood glucose, managing body weight, and reducing the risk of heart disease in patients with Type 2 diabetes [16]. However, previous clinical trial showed that compared with the Mediterranean diet, low-carbohydrate diet can better control the risk of blood glucose and heart disease in patients with T2D [17]. Huntriss’ meta-analysis showed that reducing the proportion of carbohydrate diet can better manage T2D [18]. In addition, Mohammad's survey in 2020 showed that compared with conventional diet, Mediterranean diet could not effectively reduce body mass index (BMI) and multiple risk indicators of heart disease in patients with T2D [19]. It’s worth noting that, despite the 2015meta-analysis on the effects of the Mediterranean diet on blood glucose in diabetic patients [16], this information is now eight years old, and further research may be warranted to provide updated insights.

A network meta-analysis was conducted in 2019 [11] which compared major dietary patterns, the included RCTs were published between 2006 and 2016, Only 5 articles on the Mediterranean diet were included. In 2021 tang et al. meta-analyzed a cohort study correlating the Mediterranean diet with mortality in patients with cardiovascular disease and showed that Mediterranean diet improved survival in people with a history of CVD, But there is no validation of specific serum indicators [20]. Lotfi et al. [21] conducted a meta-analysis of prospective cohort studies of the Mediterranean diet, five-year weight change, and risk of overweight and obesity, which showed that the Mediterranean diet was inversely associated with five-year weight change and risk of overweight/obesity, but the present study meta-analysed the weight metrics of the cohort studies only, and has not yet that of the randomized cohort trial and other metrics [21]. Sarsangi et al. [22] meta-analysis of a prospective cohort study of the Mediterranean diet and risk of T2D showed that the Mediterranean diet reduces the risk of T2D, but specific indicators of the Mediterranean diet in patients with T2D have not yet been analyzed [22].

Therefore, it is not clear whether the Mediterranean diet is better at controlling glycemic, weight and reducing the risk of heart disease in people with T2D than other diets.This study conducted a meta-analysis of RCTs of Mediterranean diet in patients with T2D, so as to more comprehensively grasp the effects of Mediterranean diet on cardiovascular risk factors, glycemic control, and body weight in patients with diabetes, and provide a more effective basis for clinical practice.

## Methods

This meta-analysis was conducted in accordance with the statement of the Preferred Reporting Items for Systematic reviews and Meta-Analyses (PRISMA). It was registered on the INPLASY website (Registration number: INPLASY 202160096).

### Literature research

A systematic literature search was conducted in three English databases (PubMed, EMBASE, Cochrance Central Register of Controlled Trials, Web of Science) and four Chinese databases (Chinese Biomedical Literature Database, Wanfang Data, Chinese National Knowledge Infrastructure, and Chinese Science and Technology Periodical Database) from inception to December 2023. The search terms comprising Medical Subject Heading (MeSH), free text and word variants were as follows: (1) Diet, Mediterranean, combined explored versions of Medical Subject Headings (Mesh) ‘Mediterranean Diet’ ‘Diets, Mediterranean’ OR ‘Mediterranean Diets’; (2) Diabetes Mellitus, Type 2 combined explored versions of Medical Subject Headings(Mesh) ‘Diabetes Mellitus, Noninsulin-Dependent’ OR ‘Diabetes Mellitus, Type II’ ‘NIDDM’ OR ‘Diabetes Mellitus, Noninsulin Dependent’ OR ‘Slow-Onset Diabetes Mellitus’ OR ‘Diabetes Mellitus, Adult Onset’ and so on.We combined these terms as follows: (1) and (2).

### Study selection

Two researcher assessed literature eligibility independently (XZ, WZ). Cohen’s kappa statistics were used to assess the degree of agreement between the two reviewers. Any discordance opinions were resolved by a third researcher (XW). The selection criteria were as follows: (1) Participants comprised with already diagnosed T2D and aged ≥18 years, (2) Parallel or cross-over RCT, (3) The period of Mediterranean diet intervention at least lasted for 6 months, (4) Results reported to evaluate the effect of the Mediterranean diet including cardiovascular risk factors or HbA1c at least, (5) Studies published in English and Chinese. Exclusion criteria:type 1 diabetes and gestational diabetes. Observational studies, reviews, letters, case reports and news were excluded. All the articles eligible were identified by screening the titles or abstracts and then reviewing the full-text for further review.

### Data extraction

The following information was extracted by two researchers independently from the eligible studies: the first author information, year of publication, country, the characteristics of participants (number/age/and during of T2D), period of intervention, content of Mediterranean diet intervention and control intervention, and main study finding. The main results were classified into three categories:(1) cardiovascular risk factors including total cholesterol(TC), high-density lipoprotein cholesterol(HDL), low-density lipoprotein cholesterol(LDL), systolic blood pressure(SBP) and diastolic blood pressure(DBP). (2) glycemic control including HbA1c and fasting plasma glucose(FPG), (3) weight loss including BMI, waist circumference(WC) and body weight.

### Risk of bias assessment

The risk of bias of RCTs was assessed using version 2 of the Cochrane risk-of-bias tools for randomized trials (ROB 2), which included six categories: Randomization Process, Bias due to deviations from intended interventions, Bias due to missing data, Bias in measurement of outcomes, Bias in selection of the reported result and overall. The above six risk of bias domains were classified into three levels as a low risk ,some concerns and a high risk. The studies quality assessment was done independently by two researchers (XZ, WZ), any disagreement between the two researchers was solved by the third researcher (XW).

### Data synthesis and analysis

Data analysis were carried out using Review Manager 5.3 software. The mean differences between Mediterranean diet and control diet with 95% confidence intervals(CIs) were calculated for continuous outcomes. 95% CIs and *P*-values for some studies. We used Mediterranean diet in terms of combining statistics, Variables with inconsistent units are converted to the same units and then combined for calculation. We tested for heterogeneity using I^2^, I^2^ value <25%, 25-50%, and >50% indicate low, medium and high heterogeneity respectively. I^2^ value greater than 50% was considered substantial. Sensitively analysis and subgroup analysis were performed to explore the potential sources of high heterogeneity.

## Results

### Study selection and characteristics

**Fig. 1:**
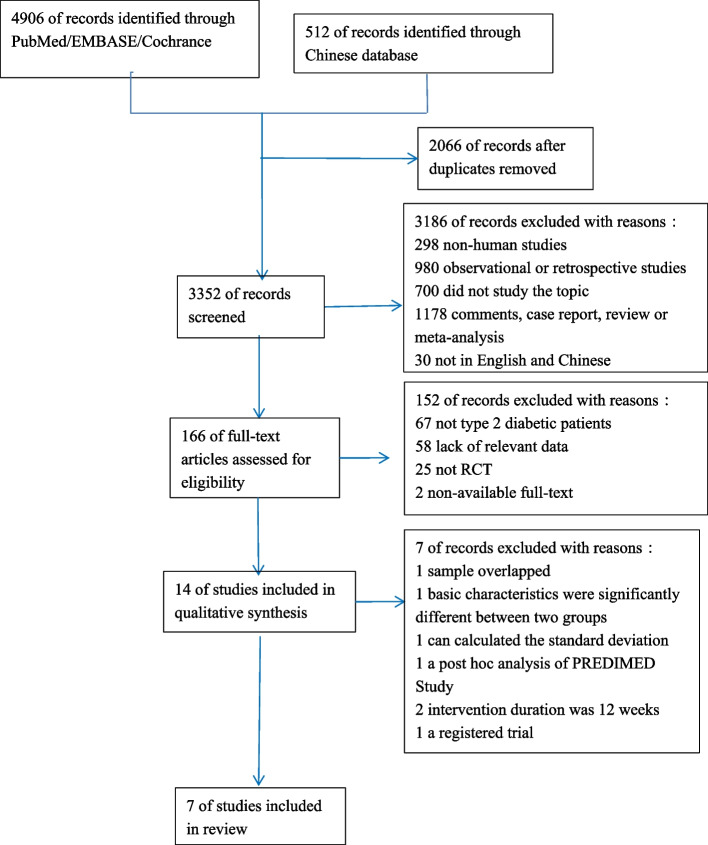
Flow diagram of literature research and study selection

A total of 5418 unique citations were identified, of which 4906 records were identified through PubMed, EMBASE, Cochrance Central Register of Controlled Trials and Cochrance Database and Web of Science, 512 records were identified through Chinese Biomedical Literature Database, Wanfang Data, Chinese National Knowledge Infrastructure. 3352 records remined after removing 2066 records duplicates. After screening title and abstract, 166 records remained for full-text review. After further evaluation of full text, 152 records were excluded for not meeting the unique criteria, the excluded due to the reasons described in Fig. 1. A total 14 studies included in qualitative synthesis, of these, 4 studies were excluded for the following reasons: One study sample overlapped [23], One study the basic characteristics were significantly different between two groups [24]. One study cannot calculated the standard deviation from the data in the original study [25], 1 study was a post hoc analysis of PREDIMED study [26]. Two studies the intervention duration was 12 weeks and two weeks, which did not meet the requirement of >6 months of intervention for this study [27, 28]. One study is a registered trial with no clinical data yet available [29]. Finally, a total of 7 studies met the inclusion criteria involving 1371 patients entered into this meta-analysis.

Table 1 summarized the basic characteristics of the 7 selected studies, all of which were RCTs and published in English. The duration of the intervention ranged from 6 months to 8.1 years. The participants’ ages ranged from 25 to 75 years during the intervention. The study samples ranged from 95 to 279. 3 trials were conducted in United State, others were conducted in Israel, Italy, Iran and Spain. All of the selected trials studied the effects of Mediterranean diet on the patients with T2D. One study compared two types of Mediterranean diet versus a control diet [17], therefore, 7 RCTs with 8 arms were included in the quantitative due to treat these arms in isolation. The control diets in these selected studies comprised high-carbohydrate diet, 2003 American Diabetes Association(ADA) diet, Low-fat diet and usual dietary. All of the selected trials comprised the basic characteristics of Mediterranean diet in the intervention diets, two trials emphasized the multifactorial intervention to improve the adherence to the Mediterranean diet (Table 1).

**Table 1:** Characteristics of included studies

First author	Year	Country	Participants	No.of cases	Age	During design	Mediterranean diet intervention	Control intervention	Available outcomes
Brehm [30]	2009	United states	Overweight/obese adults with well-controlled T2D	124 (95 completers)	38-75	1 year	High intake of olive, nuts, seeds, legumes;45% carbohydrate, 15% protein and 40% fat(with 20% MUFA, emphasizing olive and canola oils)	High-carbohydrate diet:60% carbohydrate,15% protein and 25% fat	HbA1c,fasting plasma glucose, fasting insulin, HOMA-IR, body weight, TC, LDL, HDL and blood pressure
Toobert [31]	2003	United states	Post-menopausal women with T2D	279 (245 completers)	<75	6 month	The diet recommended increased amounts of bread, root vegetables, green vegetables, legumes, and fish; less red meat (e.g, beef, lamb, and pork), substituting poultry; no day without fruit; and avoidance of butter and cream, substituting olive oil/canolaoil or commercially available olive oil/can	Usual care(no information provided)	HbA1c,triglyceride s,TC, HDL, LDL, BMI, SBP, DBP
Elhayany [17]	2010	Israel	Overweight adults with T2D	259 (179 completers)	30-65	1 year	A low-carbohydrate mediterranean diet (50-55% LGI carbohydrate, 45% fat-high in MUFA and 20% protein) A traditional mediterranean diet (50-55% LGI carbohydrate, 30% fat-high in MUFA and 15-20% protein)	American Diabetes Association diet: 50-55% carbohydrate of a mixed glycemic index,30% fat and 15-20% protein.	Weight, BMI, waist circumference, fasting plasma glucose, HbA1c, trig lycerides, TC, HDL, LDL,, fasting insulin,HOMA-IR
Maiorino [32]	2016	Italy	Adults with newly Diagnosed T2D	215 (201 completers)	Mean 52	8.1years	Rich in whole grains and vegetables,low in red meat, >30% calories from fat,30 to 50g of olive oil, <50% calories from carbohydrate	Low-fat diet:<30% calories from fat(<10% calories from saturated fat),with restricted additional fats,sweets and high-fat snacks	Weight,WC,HbA1c, plasma glucose,triglycerides, TC , HDL , LDL, SBP,DBP
Maryam [19]	2020	Iran	Adults with T2D	228	40-60	6 month	Received 8 education sections and underwent classic MD in 25-person groups in two times by nutritionist.	Usual care(no information provided)	Fasting blood glucose,HbA1c,trig lycerides, TC , HDL, LDL, SBP, DBP, BMI
Alonso-Dominguez [33]	2019	Spain	Adults with T2D	204 (185 completers)	25-70 Mean 60.6	1 year	Received brief advice about healthy eating and physical activity, and took part in a food workshop, five walks and received a smartphone application for three months	Usual care(no information provided)	Glycated haemoglobin,Postp randial glucose,Atherogeni c index, TC, HDL, LDL, Waist circumference, SBP, DBP
Toobert [34]	2011	United States	Latinas with T2D	280	Mean 57	2 year	Emphasis on vegetables, legumes,cereals, cereals, fruits, nuts, olive oil and limited fat	Usual care (no information provided)	HbA1c, BMI

### Risk of bias assessment

Of the seven RCTs, two studies were identifified as having high risk of bias [17, 34], and five as having some concerns (Brehm et al. [30], Toobert et al. [31], Maiorino et al. [32], Maryam et al. [19], Alonso-Dominguez et al. [33]. In terms of the randomization process, four studies presented some concerns due to insufficient information on allocation concealment (Brehm et al. [30], Toobert et al. [31], Elhayany et al. [17], Maryam et al. [19]. All of the seven studies were considered to have some concerns to the risk of bias from assignment to interventions due to none of the seven studies reported the success of blinding of participants and personnel for dietary intervention. Two study had high risk of bias for missing outcome data because of a high proportion of participant loss [17, 34]. Another two studies were considered to have some concerns due to insufficient information provided about the blinding of outcome assessors (Toobert et al. [31]; Maryam et al. [19]. All of the seven studies were considered to have some concerns in the bias of reported results due to the absence of registered protocols (Table 2).

**Table 2:** Risk of bias of included studies

Study, Year	Randomization process	Bias due to deviations from intended interventions	Bias due to missing data	Bias in measurement of outcomes	Bias in selection of the reported result
Brehm, 2009 [30]	Some concerns	Some concerns	Low risk of bias	Low risk of bias	Some concerns
Toobert, 2003 [31]	Some concerns	Some concerns	Low risk of bias	Some concerns	Some concerns
Elhayany, 2010 [17]	Some concerns	Some concerns	High risk of bias	Low risk of bias	Some concerns
Maiorion, 2016 [32]	Low risk of bias	Some concerns	Low risk of bias	Low risk of bias	Some concerns
Maryam, 2020 [19]	Some concerns	Some concerns	Low risk of bias	Some concerns	Some concerns
Alonso-Domínguez, 2019 [33]	Low risk of bias	Some concerns	Some concerns	Low risk of bias	Some concerns
Toobert, 2011 [34]	Low risk of bias	Some concerns	High risk of bias	Low risk of bias	Some concerns

### Cardiovascular risk factors

**Fig. 2:**
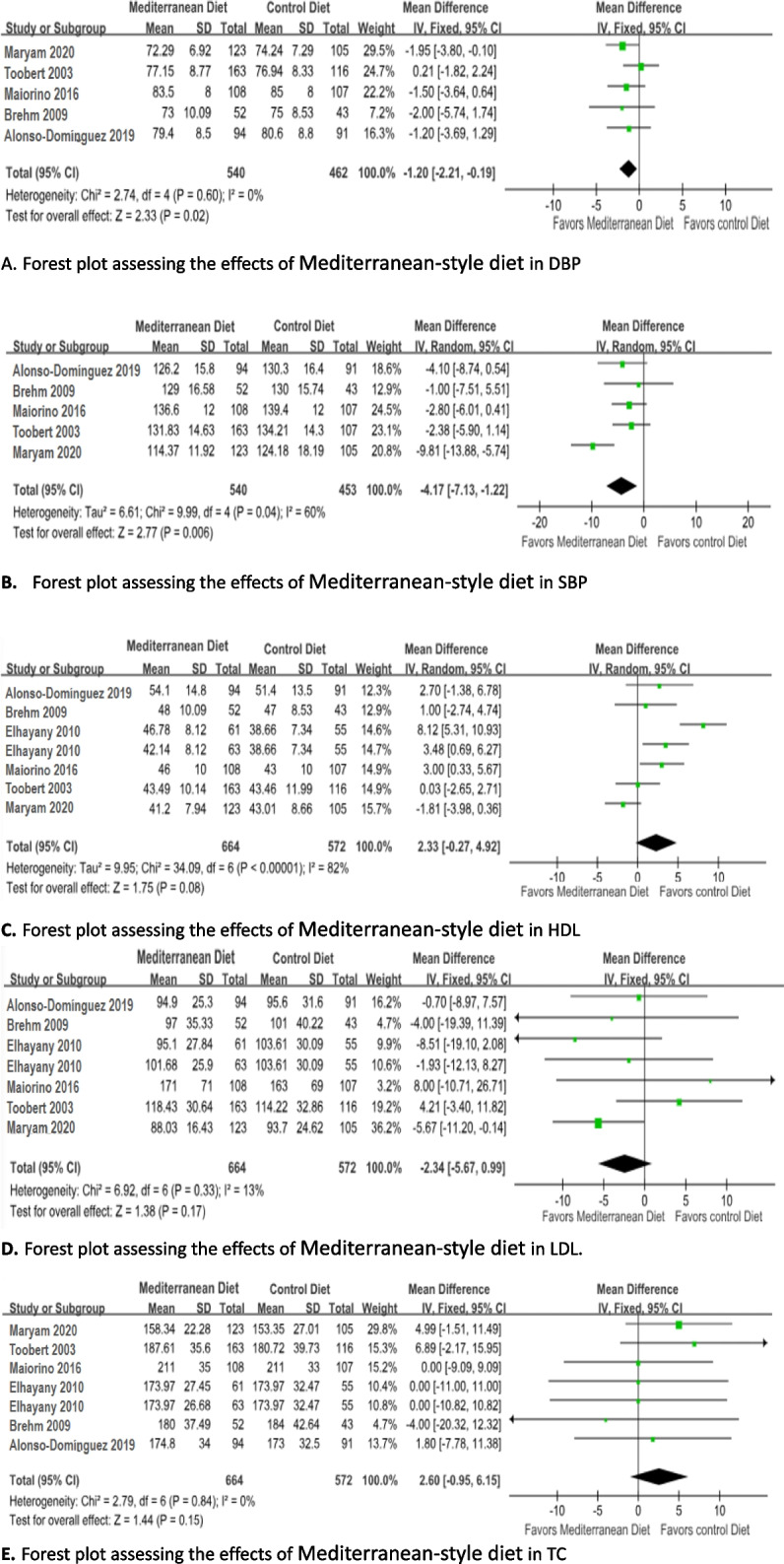
Forest plot assessing the effects of Mediterranean-style diet in DBP(**A**), SBP(**B**), HDL(**C**) , LDL(**D**) and TC(**E**). Alonso-Dominguez 2019 is the comparison between mediterranean diet and usual care; Brehm 2009 is the comparison between mediterranean diet and High-carbohydrate diet; Elhayany 2010 is the comparison between two mediterranean diets and American Diabetes Association diet; Maiorino 2016 is the comparison between mediterranean diet and Low-fat diet; Toobert 2003 is the comparison between mediterranean diet and usual care; Zahedi 2020 is the comparison between mediterranean diet and usual care

Five studies reported the SBP and DBP data that could be pooled in the analysis. The Mediterranean diet significantly decreased the level of DBP by fixed effects model (MD = -1.20; 95% CI: -2.21 to -0.19, *I*^*2*^= 0 %; *P* = 0.02) (Fig. 2a) and could significantly reduce the level of SBP by random effects model (MD = -4.17; 95% CI: -7.13 to -1.22; I^2^= 60%; *p* = 0.006) (Fig. 2b) through compared with control diet.

Changes in HDL and LDL were pooled for six studies with seven arms (*n* = 1181). Meta-analysis showed a trend toward reducing the level of LDL (MD = -2.34; 95% CI:-5.67 to 0.99; I^2^ = 13%; *p* = 0.17) by fixed effects model (Fig. 2c) and increasing the level of HDL (MD = 2.33; 95% CI: -0.27 ~ 4.92; I^2^ = 82%; *p* = 0.08) (Fig. 2d), but the beneficial effects of Mediterranean diet compared with control diets were not statistically significant. Moreover, Mediterranean diet could not reduce the level of TC compared with control diets (MD = 2.60; 95% CI: -0.95 ~ 6.15; I^2^ = 0%; *p* = 0.15) (Fig. 2e).

### Glycemic control

**Fig. 3:**
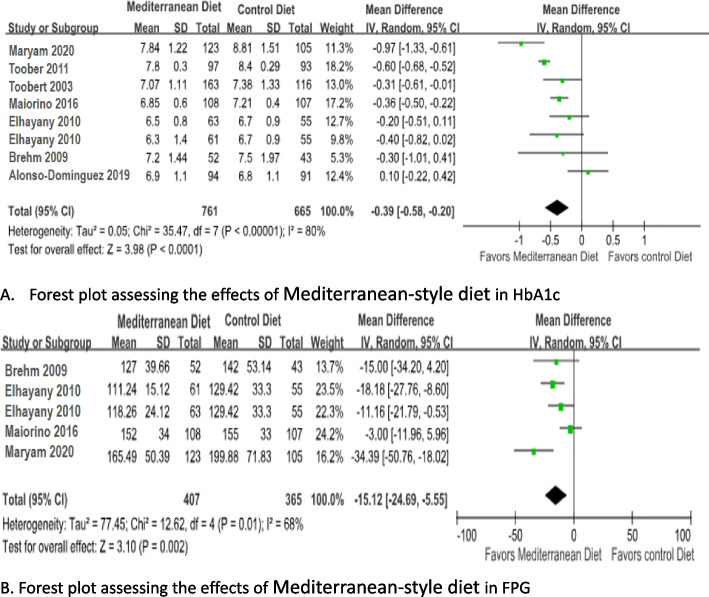
Forest plot assessing the effects of Mediterranean-style diet in HbA1c(**A**) and FPG(**B**). Alonso-Dominguez 2019 is the comparison between mediterranean diet and usual care; Brehm 2009 is the comparison between mediterranean diet and High-carbohydrate diet; Elhayany 2010 is the comparison between two mediterranean diets and AmericanDiabetes Association diet; Maiorino 2016 is the comparison between mediterranean diet and Low-fat diet; Toobert 2003 is the comparison between mediterranean diet and usual care; Zahedi 2020 is the comparison between mediterranean diet and usual care; Toobert 2011 is the comparison between mediterranean diet and usual care

The data for the change in HbA1c level were pooled from seven studies with eight arms (*n* = 1371). Meta-analysis showed that the Mediterranean diet was associated with a significant reduction in HbA1c compared with the control diet (MD = -0.39; 95% CI: -0.58 to -0.20; I^2^= 80%, *p*<0.001) by random effects model (Fig. 3a).

Four studies with five arms (*n* = 717) measured the changes in FPG. Meta-analysis showed that subjects who accepted Mediterranean diet had decreased FPG levels (MD= -15.12 mg/dl, 95% CI: -24.69 ~ -5.55; *p* = 0.002) compared with those who accepted control diet (Fig. 3b) by random effects model due to the high heterogeneity (I^2^= 68%) .

### Weight loss

**Fig. 4:**
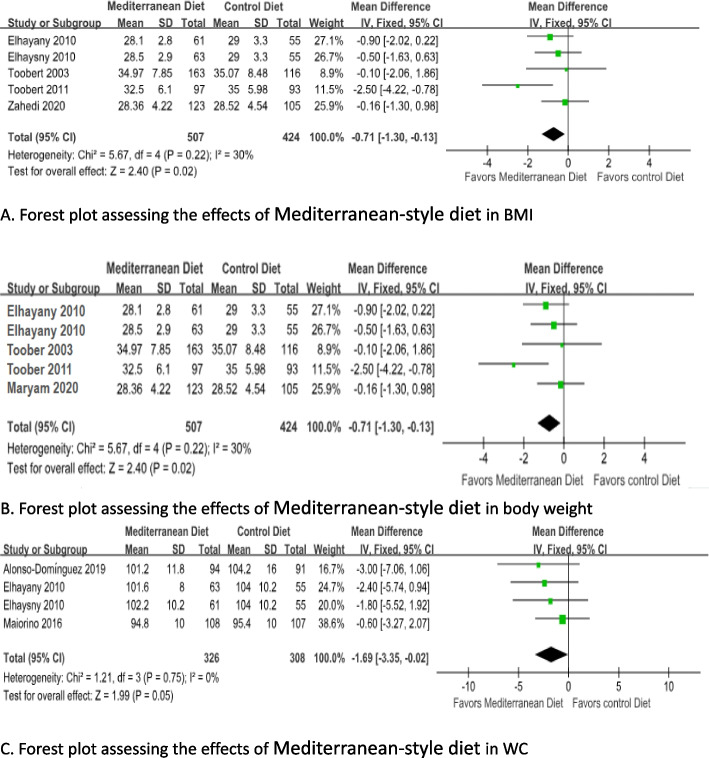
Forest plot assessing the effects of Mediterranean-style diet in BMI(**A**), body weight(**B**) and WC(**C**). Alonso-Dominguez 2019 is the comparison between mediterranean diet and usual care; Brehm 2009 is the comparison between mediterranean diet and High-carbohydrate diet; Elhayany 2010 is the comparison between two mediterranean diets and AmericanDiabetes Association diet; Maiorino 2016 is the comparison between mediterranean diet and Low-fat diet; Toobert 2003 is the comparison between mediterranean diet and usual care; Zahedi 2020 is the comparison between mediterranean diet and usual care

Changes in BMI levels were pooled for four studies with five arms. Subjects who consumed Mediterranean diet (*n* = 507) showed statistically significant decline in BMI (MD = -0.71; 95% CI: -1.30 to -0.78; *p* = 0.02) compared with subjects with control die t(*n* = 369) by using a fixed effects model (I^2^= 30%) (Fig. 4a).

Only three studies with four arms (*n* = 489) reported data on body weight, the reduction in body weight was not statistically significant trough Mediterranean diet compared with control diet (MD = -1.24; 95% CI: -3.23 to 0.74; *p* = 0.22) by fixed effects model due to no heterogeneity was observed (I^2^= 0.0%) (Fig. 4b).

In additional, three studies with four arms (*n* = 579) measured the WC and were pooled in the analysis. There was a statistically significant decline in WC level between Mediterranean diet and control diets by a fixed effects model (MD = -1.69; 95% CI:-3.35 to -0.02; I^2^= 0 %; *p* = 0.05) (Fig. 4c).

### Publication bias

The funnel plot showed some evidence of potential publication bias for HbA1c, FPG, SDP and HDL, there were no evidence of substantial publication bias for the other measurement index (BMI, body weight, WC, BDP, TC and LDL).

### Sensitivity analysis and subgroup

**Fig. 5:**
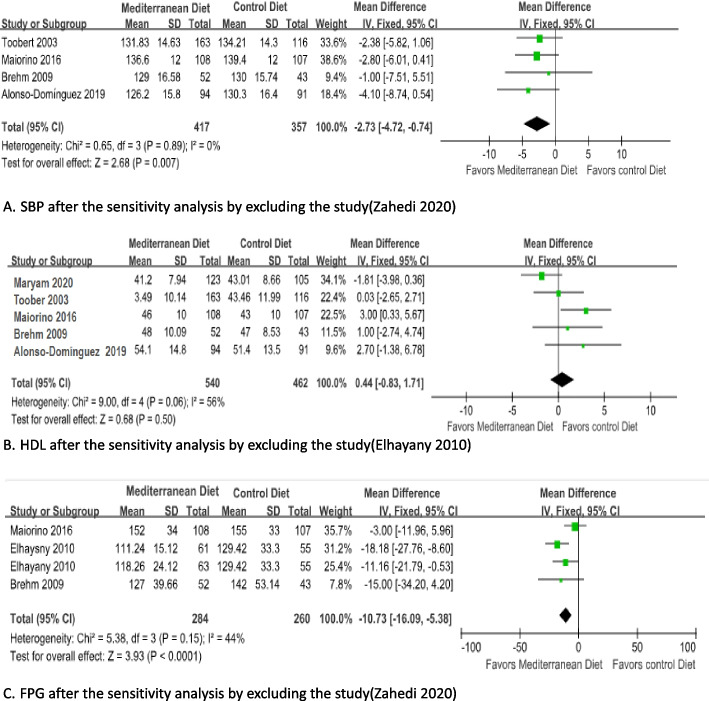
SBP(**A**), HDL(**B**), FPG(**C**) after the sensitivity analysis by excluding study. Alonso-Dominguez 2019 is the comparison between mediterranean diet and usual care; Brehm 2009 is the comparison between mediterranean diet and High-carbohydrate diet; Elhayany 2010 is the comparison between two mediterranean diets and American Diabetes Association diet; Maiorino 2016 is the comparison between mediterranean diet and Low-fat diet; Toobert 2003 is the comparison between mediterranean diet and usual care; Zahedi 2020 is the comparison between mediterranean diet and usual care

In the sensitivity analysis, the pooled effects can not alter by omission of any individual study from the meta-analysis in the level of HbA1c. When we excluded one study with Zahedi 2020 in SBP, the heterogeneity was changed (I^2^= 0 %), the effect estimate was unchanged by the Fixed effects model (MD -2.73; 95% CI -4.72 ~ -0.74; I^2^ = 0 %; *P* = 0.007) (Fig. 5). When we excluded one study with Zahedi 2020 in FPG, the heterogeneity was changed (I^2^= 44 %), the effect estimate was unchanged by the Fixed effects model (MD -10.73; 95% CI -16.09 ~ -5.38; I^2^ = 44 %; *P* < 0.001) (Fig. 5). But changes in the HDL, when we excluded one study with two arms (Elhayany 2010), the heterogeneity was changed (I^2^= 56%), the effect estimate was unchanged by the random effects model (MD 0.75; 95% CI -1.22 ~ 2.73; I^2^ = 56%; *P* = 0.46) (Fig. 5), the result show Mediterranean diet cannot improve the level of HDL compared with the control diet.

High heterogeneity were found in the primary outcomes of HbA1c and HDL, therefore the subgroup analysis was performed for further analysis. The subgroup analyses in HbA1c levels were stratified by country, number of participants, disease characteristics of participants and quality of the selected studies. The results by the subgroup analysis were similar to the primary analysis (Table 3). However, subgroup analyses of HDL according to the study population showed that heterogeneity was still high, and subgroup analyses of SBP and FPG were not possible due to the small number of included studies in the literature.

**Table 3:** Subgroup analyses for primary outcome of HbA1c

*Subgroup*	*No. of studies*	*Pooled mean differences(95% CI)*	*P for heterogeneity*	*I*^*2*^*(%)*
Country				
United states	3	-0.48[-0.71, -0.25]	0.14	50
Others	4	-0.36[-0.64, -0.07]	<0.001	80
Sample size				
< 250	4	-0.38[-0.79, 0.02]	<0.001	84
>= 250	3	-0.41[-0.63, -0.18]	<0.001	68
Characteristics				
T2D	4	-0.33[-0.44, -0.22]	0.91	0
Special adults with T2D	3	-0.49[-0.97, -0.00]	<0.001	91
Quality				
Low	4	-0.44[-0.74, -0.15]	0.02	65
High	3	-0.33[-0.62, -0.03]	<0.001	91

## Discussion

Meta-analysis of the RCTs that met the inclusion criteria showed that Mediterranean diet could better control glycemic, reduce the BMI and blood pressure in patients with T2D, but the improvement effects on HDL, LDL and TC were not obviously and needed to be further verified.

In this meta-analysis, although the Mediterranean diet did not reach statistical significance in terms of its effect on HDL and LDL compared to the control group, there was still a tendency to increase HDL and decrease LDL levels, as well as the Mediterranean diet reduced the levels of SBP and DBP, which fully validates that the Mediterranean diet reduces the risk of heart disease in patients with T2D. Studies have shown that every 0.1% decrease in HbA1c level can reduce the risk index of heart disease by 7% [35]. Intake of 10g of olive oil per day can reduce the risk of heart disease by 16% [36, 37]. Conducted a Real Life Study on the composition of each food in Mediterranean diet, the results showed that cardiovascular risk factors was reduced by 21% in the high Mediterranean diet group compared with the low Mediterranean diet group. Increasing the proportion of vegetables and fruits can make the goal of lowering LDL easier to achieve, and increasing fish intake can significantly reduce TC levels. However, in this study, the effects of Mediterranean diet on HDL and LDL have not reached statistical significance, and the TC level cannot be effectively reduced, which is somewhat different from the conclusions of previous studies, and may be partly related to the large difference in the baseline level of serum indicators (HDL/LDL/TC) of the two groups of subjects in only 6 literatures included in this study. Therefore, the effect of Mediterranean diet on HDL and LDL still needs to be further verified.

Mediterranean diet can reduce the levels of HbA1c and FPG in patients with T2D, which is consistent with the results of two meta-analyses in 2013 and 2015 [16, 38]. This may be associated with a high proportion of vegetables, monounsaturated fatty acids, nuts and fruits in Mediterranean diet. This kind of food can effectively improve insulin sensitivity and prevent production of advanced glycosylated end products and other factors [39]. However, the degree to which Mediterranean diet reduced HbA1c was strongly related to the type of diet compared. In addition, there are certain differences in the level of HbA1c reduction compared with different diet types. Compared with conventional diet, Mediterranean diet can reduce HbA1c level by 0.53 [16], but compared with low-fat diet, Mediterranean diet can only reduce HbA1c by 0.32 [40]. Moreover, the research results of Snorgaard et al [41] showed that low-fed diets achieved higher rates of diabetes remission (defined as HbA1c <6.5%) compared with control diets. Therefore, the study showed that low-fed diets could more effectively manage HbA1c in patients with age over 60 years old, while Mediterranean diet had better effect on patients with age under 60 years old. In addition, studies have shown that Mediterranean diet can reduce insulin resistance [16]. However, due to the few literatures included in this meta-analysis that included this indicator, the effect of Mediterranean diet on insulin resistance was not analyzed.

Mediterranean diet can reduce the BMI and WC of patients with T2D, which is consistent with the results of many previous studies [11, 16, 41], and may be related to the fact that Mediterranean diet is rich in a large number of vegetable, grain and other plant foods, which provide more dietary fiber and reduce the load of carbohydrates. Moreover, the research results of Hashimoto et al. [42] showed that low-fed diets can better manage body weight and fat mass compared with Mediterranean diet which may be related to the age of the research object in the included literature to some extent. However, in this meta-analysis, the effect of Mediterranean diet on the reduction of WC was not obvious, possibly because there were only 3 studies that included WC in the outcome variables in the included literatures in this meta-analysis, and the results may be biased to some extent.

Each component of the special diet of Mediterranean diet can have a great correlation with the homeostasis of diabetes, and many effects may have common physiological and pathological pathways. Mediterranean diet not only plays an obvious role in blood glucose control, weight reduction, and risk reduction of heart disease, etc. Furthermore, studies have shown that Mediterranean diet affects glucagon‐like peptide 1 [42], oxyntomodulin [43], postprandial lipemia [44], and antioxidant/ antioxidant enzymes compounds [45], gut microbiota composition and function [46, 47], branched chain aminoacid management [48] and many other aspects have certain significance. Therefore, under the synergistic effect of various components in Mediterranean diet, Mediterranean diet becomes a valuable dietary pattern for primary and secondary prevention of diabetes and gradually becomes a healthy and nutritious dietary lifestyle. However, there are still some differences in the results of group multi-action, which need to be further verified.

Therefore, in the future, scholars should pay more attention to the study of objective clinical indicators, such as heart disease risk indicators, in order to provide a more accurate theoretical basis for clinical practice. As there are various types of diabetes diets, scholars could employ Network Meta-analysis in the future to potentially yield more accurate results.

### Limitations

This meta-analysis includes seven RCTs involving a total of 1371 patients with T2D, Several limitations should be recognized. First, the participants in this study were all diabetic patients, and there was no study on non-diabetic patients. According to the survey by Carter et al. on all populations with or without diabetes, Mediterranean diet can only reduce the level of HbA1c, but its effect on FPG is not obvious [49]. Furthermore, the control group in this study included various diets, which may have a certain influence on the results of the study, because the role of different diets in different studies varies to a certain extent. Finally, the relatively insufficient number of trials limits our ability to conduct further subgroup analysis on some specific indicators.

## Conclusion

This meta-analysis provides convincing evidence for helping health administrators to identify effective dietary strategy for patients with T2D. The results reveal that Mediterranean diet has a more prominent role in management of T2D, which is effective in control blood pressure (DBP and SBP), glycemic control (HbA1c and FPG), losing weight (BMI and WC), but the beneficial effects in meliorate lipid were not statistically significant (HDL, LDL and TC) compared with control diets, which need to further verification.

### Supplementary Information

**Additional file 1.** Fourteen of studies included in qualitative synthesis, 7 of records excluded table.

## Data Availability

Data materials are real and available. Xing ZHENG should be contacted if someone wants to request the data from this study.

## Abbreviations

T2D: Type 2 Diabetes
RCTs: Randomized Controlled Trials
ROB 2: Cochrane risk-of-bias tools for randomized trials
HDL: High-density lipoprotein
LDL: Low-density lipoprotein
SBP: Systolic blood pressure
DBP: Diastolic blood pressure
FPG: Fasting plasma glucose
PRISMA: Preferred Reporting Items for Systematic reviews and Meta-Analyses
MeSH: Medical Subject Heading
WC: Waist circumference
BMI: Body mass index
CIs: Confidence intervals
