# Supplementary material for: The effects of Mediterranean diet on cardiovascular risk factors, glycemic control and weight loss in patients with type 2 diabetes: a meta-analysis
Source: BMC Nutr. 2024 Apr 19;10:59. doi: 10.1186/s40795-024-00836-y (PMC11027355; doi:10.1186/s40795-024-00836-y)
Supplement: Supplementary file 1 — Additional file 1. Fourteen of studies included in qualitative synthesis, 7 of records excluded table. [file 40795_2024_836_MOESM1_ESM.docx]

14 of studies included in qualitative synthesis，7 of records excluded table：

| First author | year | number of participants | duration of intervention | outcome | reason for excluding | available outcomes |
| --- | --- | --- | --- | --- | --- | --- |
| Esposito | 2009 | 215 overweight people with newly diagnosed type 2  Diabetes；  Mediterranean-style diet (n 108) or a low-fat diet (n 107) | 4 years | Weight（weight, body mass index, waist circumference）;  Glycemic control(HbA1c, plasma glucose level, Serum insulin level, HOMA of insulin level, HOMA of insulin sensitivity, Adiponectin level);  Coronary risk factors(Total cholesterol level, HDL cholesterol level, Triglyceride level, Systolic BP, Diastolic BP) | sample overlapped | Weight（weight, body mass index, waist circumference）;  Glycemic control(HbA1c) ;  Coronary risk factors(Total cholesterol level, HDL cholesterol level, Triglyceride level, Systolic BP, Diastolic BP) |
| Monlezun | 2015 | 27 patients with T2D be  tween the control and GCCM arms  Mediterranean-style diet (n= 18) or control diet (n= 9); | 6 month | Biometric(HbA1c)  BP(SBP,DBP)  Lipids( Total cholesterol level, HDL cholesterol level, LDL cholesterol level, Triglyceride level)  HR  BMI  Psychometric(Mostly believe can eat correct portions, Eat vegetable most nights per week, Eat fruits most nights per week, Mostly use nutrition panel for food choices) | the basic characteristics were significantly different between two groups | Biometric(HbA1c)  BP(SBP,DBP)  Lipids( Total cholesterol level, HDL cholesterol level, LDL cholesterol level, Triglyceride level)  BMI |
| Pérez-Ferre | 2015 | A total of 260 women with prior GDM  Mediterranean-style diet (n= 130) or control diet (n= 130);  A total of 237 women  completed the three-year follow-up (126 in the intervention group and 111 in the control group) | Three years | BMI,WC, FP glucose, FP insulin, HOMA-IR, SBP, DBP, Total cholesterol, HDL cholesterol, LDL cholesterol, Triglyceride,Apo lipoprotein B, ACR, HbA1c-1FCC, HbA1c, Physical activity score >1, Nutrition score >5, Low glycemic index score >2, Unsaturated fat score >2, Saturated fat score >3, Healthy fat score >4, Low fat score >2 | cannot calculated the standard deviation from the data in the original study | BMI,WC,SBP, DBP, Total cholesterol, HDL cholesterol, LDL cholesterol, Triglyceride,HbA1c, |
| Basterra-Gortari | 2019 | 3,230 participants with type 2 diabetes at baseline ( Med-EatPlan + EVOO n = 1158, Med-EatPlan + Nuts n = 1017, Control eating plan n= 1055);  A total of 1240 participants  Included in glucose-lowering medication initiation analyses( Med-EatPlan + EVOO n = 477, Med-EatPlan + Nuts n = 394, Control eating plan n= 369) | 3.2years and 5.1 years | free of glucose-lowering medications,  the probability of requiring insulin therapy | a post hoc analysis of PREDIMED study | none |
| Gardner | 2022 | 42 participants with PreD and T2DM  11 with PreD Randomized to Keto-Med diet sequence,  10 with T2DM Randomized to Keto-Med diet sequence,  12 with PreD Randomized to Med-Keto diet sequence,  9 with PreD Randomized to Med-Keto diet sequence | 12 weeks | HbA1c values, fasting glucose levels, weight,  HDL cholesterol levels  LDL cholesterol levels  Fasting insulin, triglyceride, ALT  quality of life, perceived cognitive function,  wellness, and gastrointestinal symptoms | the intervention duration was 12 weeks | HbA1c values,weight,  HDL cholesterol levels,  LDL cholesterol levels, triglyceride |
| Rein | 2022 | 23 adults with newly diagnosed T2DM  PPT-MED (n = 11)  MED-PPT (n = 12) | two 2-week-long dietary interventions | Anthropometric measurements( weight, BMI, Waist circumference, Body fat)  Lipid profile( Serum total cholesterol, Serum LDL cholesterol, Serum HDl cholesterol, Serum triglycerides)  Glucose biomarkers( HbA1c, Fructosamine, Fasting plasma glucose) | two 2-week-long | Anthropometric measurements( weight, BMI, Waist circumference, Body fat)  Lipid profile( Serum total cholesterol, Serum LDL cholesterol, Serum HDl cholesterol, Serum triglycerides)  Glucose biomarkers( HbA1c) |
| Papamichou | 2022 | 120 adults aged 20e75 with a body mass index (BMI) of 20e35 kg/m2 and  T2DM | 24 weeks | The primary outcome is changes in HbA1c from baseline to 12 and 24 weeks.,  Secondary outcomes include  fasting blood glucose, insulin, blood lipids, weight loss, insulin resistance index (HOMA), Glucagon-like peptide 1 (GLP-1) and high-sensitivity C- reactive protein (hs-CRP). Data on medical history, anthropometry, wellbeing, MedDiet adherence and satiety will be measured at a private clinic via self-report questionnaires at baseline, 6, 12 and 24 weeks. Additionally, specimens (blood, urine and stool) will be collected at all time points for future omics analysis. | a registered trial with no clinical data yet available | HbA1c  blood lipids  weight loss |
